# Supplementary material for: Therapeutic potential of hookworm proteins in promoting regulatory immune responses to modulate Trypanosoma cruzi induced liver inflammation and oxidative stress
Source: Mem Inst Oswaldo Cruz. 2026 Feb 13;121:e250123. doi: 10.1590/0074-02760250123 (PMC12904142; doi:10.1590/0074-02760250123)
Supplement: Supplementary material [file 1678-8060-mioc-121-e250123-s.pdf]

Mem Inst Oswaldo Cruz, Rio de Janeiro, Vol. *121*: e250123, 2026

TABLE I

Cruzi and GAPDH primers and probes sequence

Trypanosoma cruzi 166 base pair (bp) segment- based on TcZ1 and TcZ2 primers	
Cruzi 1 (Forward)	5’ ASTCGGCTGATCGTTTTCGA 3’
Cruzi 2 (Reverse)	5’ AATTCCTCCAAGCAGCGGATA 3’
Cruzi 3 (Probe)	5’ 6-FAM CACACACTGGACACCAA MGB 3’
Mouse housekeeping gene-GAPDH (Amplicon 124 bp)	
GAPDH Forward	5’ CAA TGT GTC CGT CGT GGA TCT 3’
GAPDH Reverse	5’ GTC CTC AGT GTA GCC CAA GAT G 3’
GAPDH Probe	5’ 6-FAM CGT GCC GCC TGG AGA AAC CTG CC MGB 3’

TABLE II

Thermo fisher TaqMan gene references

Gene	Assay ID
Arg1	Mm00475988_m1
Btg2	Mm00476162_m1
Cox2	Mm00478374_m1
Mmp9	Mm00442991_m1
Nfe2l2	Mm00477784_m1
NfK-B	Mm00476361_m1
Nos2	Mm00440502_m1
Stat-1	Mm00439518_m1

TABLE III

Antibodies for immunophenotyping

Reagent	Fluorochrome	Vendor	Cat #
CD11b	BV711	BD Biosciences	563168
CD11c	BUV661	BD Biosciences	750482
CD25	BB790	BD Biosciences	624296
CD3	APC/Fire™	Biolegend	100268
CD4	BV605	BD Biosciences	563151
CD45	BB700	BD Biosciences	566439
CD8a	BUV615	BD Biosciences	613004
F4/80	BB660-P2	BD Biosciences	624295
I-A/I-E	AF700	Biolegend	107622
Ly6c	PE-CF594	BD Biosciences	562728
Ly6G	Superbright 704	Cytek	67-9668-82
Viability	ViaDye Red fixable	Cytek	R7-60008

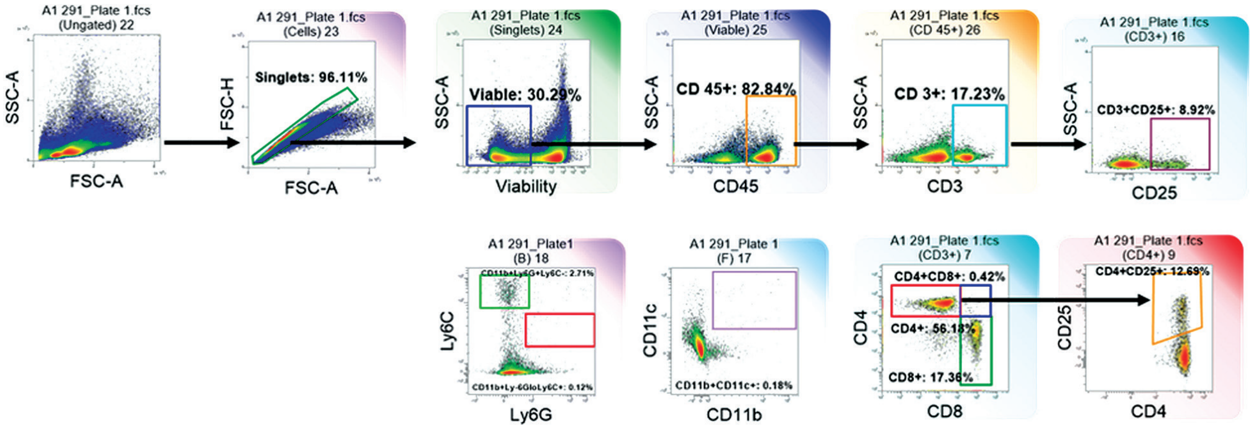

Gating strategy for splenic T cell and myeloid populations in *Trypanosoma cruzi*-infected mice. Forward scatter (FSC) vs side scatter (SSC) was used to gate on lymphocyte and myeloid populations, followed by doublet discrimination (FSC-A vs FSC-H) and viability exclusion. Total leukocytes were identified as $CD45^+$ cells. T cells were defined as $CD3^+$, with $CD4^+$ and $CD8^+$ subsets gated accordingly. Myeloid populations were identified within the $CD11b^+$ gate: $CD11b^+CD11c^+$ dendritic cells, $CD11b^+Ly6G^{lo}Ly6C^+$, and $CD11b^+Ly6G^+Ly6C^-$.
